# The spread of *bla*_OXA-48_ and *bla*_OXA-244_ carbapenemase genes among *Klebsiella pneumoniae*, *Proteus mirabilis* and *Enterobacter* spp. isolated in Moscow, Russia

**DOI:** 10.1186/s12941-015-0108-y

**Published:** 2015-11-02

**Authors:** Nadezhda K. Fursova, Eugeny I. Astashkin, Anastasia I. Knyazeva, Nikolay N. Kartsev, Ekaterina S. Leonova, Olga N. Ershova, Irina A. Alexandrova, Natalia V. Kurdyumova, Svetlana Yu. Sazikina, Nikolay V. Volozhantsev, Edward A. Svetoch, Ivan A. Dyatlov

**Affiliations:** State Research Center for Applied Microbiology and Biotechnology, Obolensk, 142279 Moscow Region, Russia; The Burdenko Neurosurgery Institute, Moscow, 125047 Russia

**Keywords:** *Enterobacteriaceae*, *Klebsiella pneumoniae*, *Proteus mirabilis*, *Enterobacter aerogenes*, *Enterobacter cloacae*, OXA-48-like carbapenemase, Hospital pathogens, Antibacterial resistance, Horizontal gene transfer

## Abstract

**Background:**

The spread of carbapenemase-producing *Enterobacteriaceae* (CPE) is a great problem of healthcare worldwide. Study of the spread for *bla*_OXA-48-like_ genes coding epidemically significant carbapenemases among hospital pathogens is important for the regional and global epidemiology of antimicrobial resistance.

**Methods:**

Antibacterial resistant isolates of *Klebsiella pneumoniae* (n = 95) from 54 patients, *P.mirabilis* (n = 32) from 20 patients, *Enterobacter aerogenes* (n = 6) from four patients, and *Enterobacter cloacae* (n = 4) from four patients were collected from January, 2013 to October, 2014 in neurosurgical intensive care unit (ICU) of the Burdenko Neurosurgery Institute, Moscow. Characteristics of the isolates were done using susceptibility tests, PCR detection of the resistance genes, genotyping, conjugation, DNA sequencing, and bioinformatic analysis.

**Results:**

Major strains under study were multi drug resistant (MDR), resistant to three or more functional classes of drugs simultaneously—98.9 % *K. pneumoniae*, 100 % *P.mirabilis*, one *E.aerogenes* isolate, and one *E.cloacae* isolate. Molecular-genetic mechanism of MDR in *K.pneumoniae* and *P.mirabilis* isolates were based on carrying of epidemic extended-spectrum beta-lactamase *bla*_CTX-M-15_ gene (87.2 and 90.6 % accordingly), carbapenemase *bla*_OXA-48-like_ gene (55.3 and 23.3 % accordingly), and class 1 (54.8 and 31.3 % accordingly) and class 2 (90.6 % *P.* *mirabilis*) integrons. The *bla*_OXA-48-like_-positive *K.* *pneumoniae* were collected during whole two-year surveillance period, while *P.* *mirabilis* and *Enterobacter* spp. carrying *bla*_OXA-48-like_ genes were detected only after four and 18 months after the research start, respectively. The *bla*_OXA-48-like_ gene acquisition was shown for *P.* *mirabilis* isolates collected from five patients and for *E.* *cloacae* isolate collected from one patient during their stay in the ICU, presumably from *bla*_OXA-48-like_-positive *K.* *pneumoniae.* The source of the *bla*_OXA-244_ gene acquired by *E.* *aerogenes* isolates and the time of this event were not recognized.

**Conclusions:**

The expanding of CPE in the surveyed ICU was associated with the spread of *bla*_OXA-48_ and *bla*_OXA-244_ carbapenemase genes documented not only among *K.pneumoniae*, well-known bacterial host for such genes, but among *P.mirabilis*, *E.aerogenes*, and *E.* *cloacae.*

## Background

One of the main problems of healthcare worldwide is hospital-acquired infections (HAI) caused by multi drug resistant (MDR) pathogens [1, 2] including carbapenemase-producing *Enterobacteriaceae* (CPE) [3]. The spread of CPE over the last decades is a great danger because carbapenems are the last treatment options for infections caused by MDR bacteria. Recently carbapenemases belonged to three Ambler-classes of beta-lactamases [4] were reported from CPE isolated in Russia: the KPC-type (class A), the NDM-type (class B), and the OXA-48-type (class D) [5]. The OXA-48 carbapenamase is enzyme that was first identified in *Klebsiella pneumoniae* clinical strains isolated in Turkey, 2003 [6]. Currently OXA-48-producers are described among many species of enterobacteria: *K.* *pneumoniae*, *Escherichia coli*, *Enterobacter cloacae*, *Citrobacter freundii*, *Serratia marcescens*, *Morganella morganii* and *Proteus mirabilis* [7–9]. The endemic areas of OXA-48-producers are Turkey, the North Africa and India. Nosocomial outbreaks caused by OXA-48-producers are registered in many European countries including France, Germany, Switzerland, Spain, Holland, and Great Britain. Sporadic cases of such infections are observed in North and South America, China, Australia, and in the Middle East countries [10]. Wide spread of *bla*_OXA-48_-like genes among enterobacteria is explained by their localization on the 62 kb conjugative plasmid belonged to IncL/M incompatibility group. Such plasmids are characterized by a high rate of transfer and a wide range of hosts. Their high conjugative potential is due to inactivation of *tir* gene coding the repressor of conjugative plasmid transfer, by the inserting of the composite transposon Tn1991/Tn1991.2 carrying the *bla*_OXA-48-like_ gene [11]. In several studies is shown that *bla*_OXA-48_-carrying plasmids provide the ability of both clonal and horizontal transfer [7]. There are several variants of *bla*_OXA-48-like_ genes coding enzymes differ from each other on some amino acid substitutions [12]. For instance, the *bla*_OXA-244_ gene discovered in Spain in 2013 differs from the *bla*_OXA-48_ gene in a single nucleotide substitution A640G that result in a single amino acid substitution Arg214Gly [13]. It should be noted that carbapenemase activity of all OXA-48-like enzymes are low, so they cannot provide the high levels of carbapenem resistance without additional contribution of other factors such as porin mutations resulting in decrease of cell wall permeability [14].

In this paper we present phenotypic and genotypic characterization of antibacterial resistant *K.* *pneumoniae*, *Proteus mirabilis*, *Enterobacter aerogenes*, and *E.* *cloacae* isolates carrying *bla*_OXA-48_ and *bla*_OXA-244_ carbapenemase genes, and discuss *K.pneumoniae* as possibly source for *bla*_OXA-48-like_ genes spreading in the ICU.

## Methods

### Bioethical requirements

The materials used in the work do not contain personal data of patients, because of clinical isolates were marked without name, date of birth, address, number of the disease history, personal documents and other personal materials. At the same time, in accordance with the Requirements of the Russian Federation Bioethical Committee, each patient signed a contract with hospital at admission to the clinic. The contract contained consent to treatment and laboratory examination, including a detailed investigation using instrumental methods.

### Bacterial isolates

Antibacterial resistant hospital isolates of *Klebsiella pneumoniae* (n = 95) from 54 patients, *Proteus mirabilis* (n = 32) from 20 patients, *Enterobacter aerogenes* (n = 6) from 4 patients, and *Enterobacter cloacae* (n = 4) from 4 patients with mechanical ventilation in neurosurgical intensive care unit (ICU) of the Burdenko Neurosurgery Institute, Moscow, were collected from January, 2013 to October, 2014. Bacterial cultures were grown on the Nutrient Medium No. 1 (SRCAMB, Obolensk, Russia), Luria–Bertani (LB) broth (Difco, USA) and Muller-Hinton broth (Himedia, India) at 37 °C. Bacterial isolates were stored in 10 % glycerol at minus 70 °C.

### Bacterial identification

Bacterial identification was done by Vitek-2 Compact (BioMerieux, France) and MALDI-TOF Biotyper (Bruker Daltonik, Germany).

### Susceptibility to antibacterial agents

Minimal inhibitory concentrations (MICs) of antibacterials were determined by Vitek-2 device (BioMerieux, France) using VITEK-2 AST N-101 and AST N-102 cards: amoxicillin/clavulanic acid (AMC), ampicillin/sulbactam (SAM), cefuroxime (CXM), cefoxitin (FOX), cefotaxime (CTX), ceftriaxone (CRO), ceftazidime (CAZ), cefoperazone/sulbactam (CFS), cefepime (FEP), ertapenem (ETP), imipenem (IPM), meropenem (MEM), tetracycline (TET), tigecycline (TGC), ciprofloxacin (CIP), chloramphenicol (CHL), gentamicin (GEN), tobramycin (TOB), amikacin (AMK), trimethoprim (TMP), trimethoprim/sulfamethoxazole (SXT), nitrofurantoin (NIT), and colistin (CST). Results were interpreted according to the 2014 European Committee on Antimicrobial Susceptibility Testing Recommendations (http://www.eucast.org/clinical_breakpoints/). *E.coli* strains ATCC 25922 and ATCC 35218 were used for quality control.

### Conjugation experiment

Conjugation was performed using previously described method [15]. Donor and recipient overnight cultures were grown under aeration conditions at 120 rpm/min during 18 h at 37 °C on LB broth containing 100 mg/L cefotaxime (Sigma, USA) for donor strain, and 200 mg/L rifampicin (Sigma, USA) for recipient strain. Five milliliters of fresh LB broth without antibiotic were inoculated by 0.05 ml overnight cultures and incubated with aeration to OD_600_ = 0.6. Then 1 ml each donor and recipient cultures were combined into one tube and incubated at 37 °C for 3 h without shaking. After that the conjugating mixture was diluted with tenfold steps and 0.1 ml was plated in triplicate on LB agar containing 100 mg/L cefotaxime and 200 mg/L rifampicin. Bacteria were incubated at 37 °C for 48 h. To determine the efficiency of conjugation the conjugation mixture was inoculated in parallel on cefotaxime + rifampicin and rifampicin. Frequency of plasmid transfer was calculated as the number of transconjugant cells per number of recipient cells.

### PCR detection of the resistance genes

PCR was performed using previously described oligonucleotide primers to detect *bla*_CTX-M_, *bla*_TEM_, *bla*_SHV_, *bla*_OXA-48-like_, *bla*_NDM_, *bla*_KPC_ beta-lactamase genes and class 1 and 2 integrons [16–21], *repA* and *traU* genes which are the genetic markers of the IncL/M plasmid [22], and the *ompK36* gene [23]. The PCR was carried using GradientPalmCycler (Corbert Research, Australia) and Thercyc cycler (DNA-Technology, Russia). PCR products were analysed by electrophoresis in 1.5 % agarose gel in a Sub-Cell GT apparatus (BioRad, USA).

### Strain genotyping

*Intra*-species genotyping of *K.* *pneumoniae*, *P.* *mirabilis*, and *Enterobacter* spp. strains was done by Random Amplified Polymorphic DNA (RAPD-PCR) using «random» primers OPA11 and Wil 2 accordingly previously described method [24].

### DNA sequencing

Cycle sequencing reactions were performed using the ABI PRISM BigDye Terminator v.3.1 kit. Purified products were analysed on an ABI PRISM 3100-Avant automated DNA Sequencer in the SINTOL Center for collective use (Moscow, Russia).

### Bioinformatic analysis

A computer analysis of DNA sequences was performed using Vector NTI9 software (Invitrogen, USA) and BLAST web resource (http://blast.ncbi.nlm.nih.gov/Blast.cgi). Class 1 and 2 integrons were analysed using INTEGRAL database (http://integrall.bio.ua.pt/?).

### GenBank accession numbers

The accession numbers of *K.pneumoniae* DNA sequences are follows: 27 *bla*_CTX-M-15_ genes [GenBank: KJ187476, KJ187477, KM058748, KM058751, KM058752, KJ469366, KC817480, KJ363319, KJ363321, KJ481796, KM085432, KM871847, KP205559, KP205560, KP205561, KP205562, KP205563, KP214528, KP214529, KP214530, KP214531, KP214532, KP214533, KP214534, KP214535, KP214536, KP214537]; one *bla*_CTX-M-3_ gene [GenBank: KP214538]; 16 *bla*_OXA-48_ genes [GenBank: KJ481797, KJ481798, KM085437, KP100448, KP100449, KP198287, KP198288, KP198289, KP198290, KP198291, KP198292, KP198293, KP198294, KP205554, KP205555, KP205556]; four *bla*_OXA-244_ genes [GenBank: KJ187475, KJ481795, KJ481799, KM058746]; six class 1 integron gene cassettes arrays [GenBank: KJ363320, KM009101, KF952266, KM009102, KC862254, KF971879]; three *ompC* genes [GenBank: KJ469369,KJ579290, KJ579291]; one *ompK36* gene [GenBank: KJ579289].

The accession numbers of *P.* *mirabilis* nucleotide sequences are follows: 10 *bla*_CTX-M-15_ genes [GenBank: KM009108, KM058749, KM058750, KC822920, KJ633803, KM085431, KM085433, KM085435, KM085436, KP271997]; one *bla*_CTX-M-3_ gene [GenBank: KM871848]; six *bla*_OXA-48_ genes [GenBank: KJ579286, KJ696733, KM058747, KP205551, KP205552, KP205553]; one *bla*_OXA-244_ gene [GenBank: KJ579285]; one class 1 integron gene cassettes array [GenBank: KM085438]; four class 2 integron gene cassettes arrays [GenBank: KJ579284, KM085439, KM085440, KP271998].

The accession numbers of *E.* *aerogenes* nucleotide sequences are follows: three *bla*_OXA-244_ genes [GenBank: KM357271, KP056309, KP205557]; the accession number of *E.* *cloacae* nucleotide sequence is one *bla*_OXA-48_ gene [GenBank: KP056311].

## Results and discussion

### Isolate sources and resistance phenotypes

Antibacterial resistant enterobacterial hospital isolates collected on the period of January, 2013 to October, 2014 from the patients with mechanical ventilation in neurosurgical intensive care unit (ICU) of the Burdenko Neurosurgery Institute, Moscow, belonged to *Klebsiella pneumoniae* (n = 95), *Proteus mirabilis* (n = 32), *Enterobacter aerogenes* (n = 6), *Enterobacter cloacae* (n = 4), *Escherichia coli* (n = 4), *Serratia marcescens* (n = 3), and *Morganella morganii* (n = 2). Carriers of OXA-48-like genes have been identified among them: 52 *K.pneumoniae*, 7 *P.mirabilis*, 3 *E.aerogenes*, and 1 *E.* *cloacae* isolates.

*K.* *pneumoniae* isolates were predominantly collected from respiratory system—endotracheal aspirate, bronchial lavage, and maxillary sinus (46.3 %), fewer isolates were obtained from the urine (40.0 %), wounds (6.3 %), lumbar and ventricular liquor (4.2 %), blood (2.1 %) and rectal swab (1.1 %). The major *P.* *mirabilis* isolates were isolated from urine (65.6 %), fewer isolates were obtained from the respiratory system (25.0 %), wounds (6.3 %) and blood (3.1 %). *E.* *aerogenes* isolates were collected from liquor (n = 5) and endotracheal aspirate (n = 1). *E.* *cloacae* isolates were collected from endotracheal aspirate (n = 3) and urine (n = 1). Major antibacterial resistant bacteria were collected from the respiratory system and from the urine and were associated with invasive equipment and instruments, namely mechanical ventilation and urinary catheters.

**Fig. 1:**
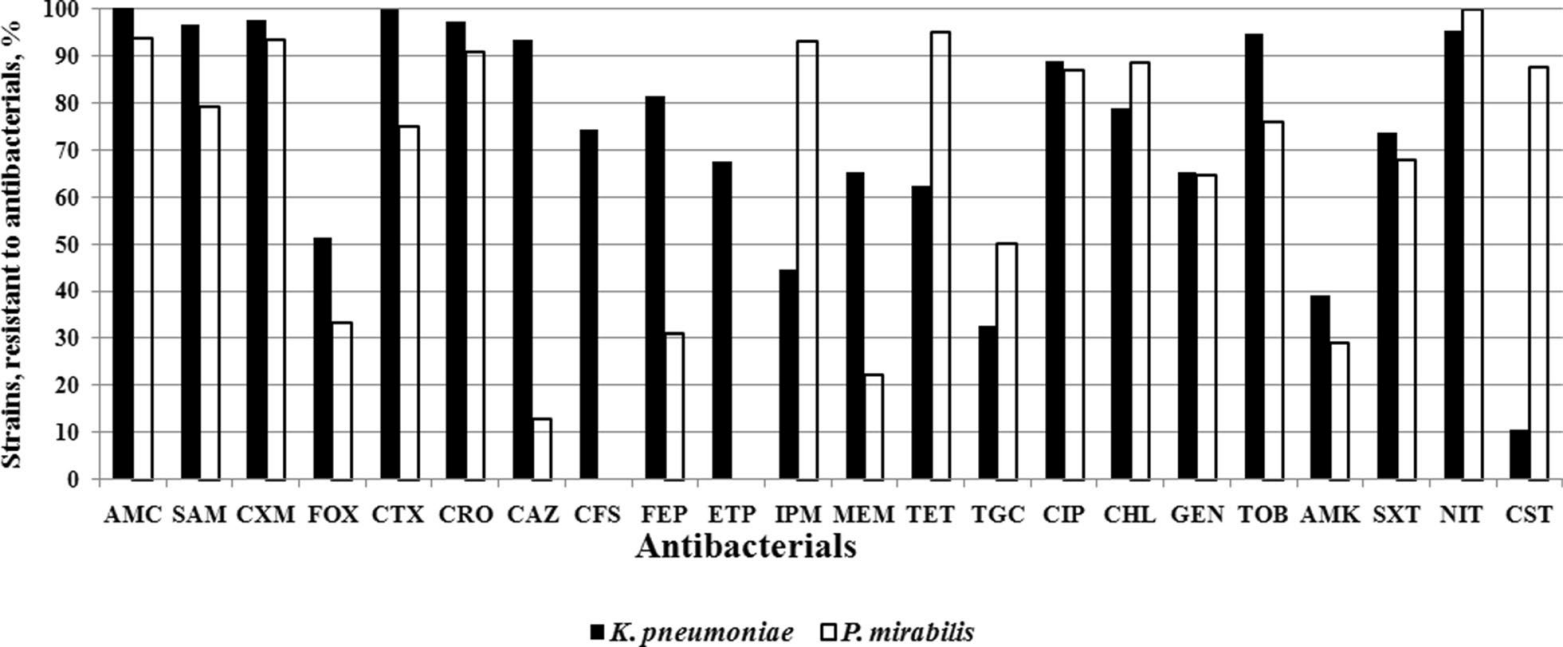
The proportion of isolates those are resistant to antibacterials: *AMC* amoxicillin/clavulanic acid, *SAM* amoxicillin/sulbactam, *CXM* cefuroxime, *FOX* cefoxitin, *CTX* cefotaxime, *CRO* ceftriaxone, *CAZ* ceftazidime, *CFS* cefoperazone/sulbactam, *FEP* cefepime, *ETP* ertapenem, *IPM* imipenem, *MEM* meropenem, *TET* tetracycline, *TGC* tigecycline, *CIP* ciprofloxacin, *CHL* chloramphenicol, *GEN* gentamicin, *TOB* tobramycin, *AMK* amikacin, *TMP* trimethoprim, *SXT* trimethoprim/sulfamethoxazole, *NIT* nitrofurantoin, *CST* colistin

**Fig. 2:**
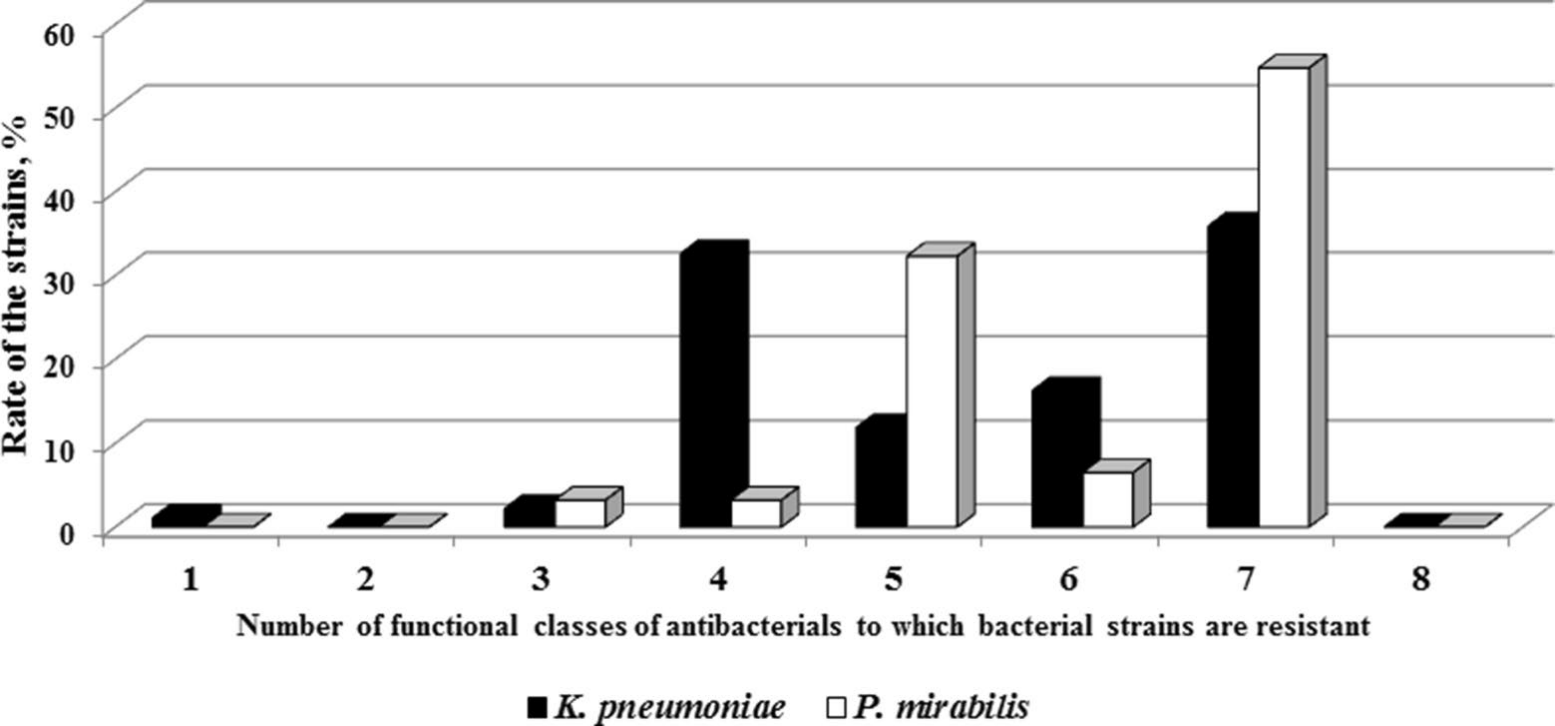
The proportion of bacterial isolates those are resistant to 1–8 functional classes of antibacterials simultaneously: beta-lactams (penicillins, cephalosporins, and carbapenems) tetracyclines, quinolones, fenicoles, aminoglycosides, polymyxins, sulfonamides, nitrofuranam

The most *K.pneumoniae* and *P.mirabilis* isolates in this study were resistant to the majority of used antibacterial agents: AMC, SAM, CXM, CTX, CRO, CIP, CHL, GEN, TOB, TMP, SXT and NIT. Additionally, the *K.* *pneumoniae* isolates were resistant to FOX, CAZ, CFS, FEP, ETP, MEM and TET, and the *P.* *mirabilis* isolates were resistant to IPM, TET, TGC and CST (Fig. 1). It is noteworthy the high proportion of the pathogens were resistant to TGC (32.4 % *K.pneumoniae* and 50.0 % *P.* *mirabilis* isolates) and to CST (87.5 % *P.* *mirabilis* isolates), that are the drugs using for the treatment of severe hospital infections. So, only one antibiotic, CST remains good activity against *K.pneumoniae* (89.5 % isolates), and only three drugs, CFS (100 % isolates), ETP (100 % isolates) and CAZ (87.1 % isolates) were effective against *P.* *mirabilis* (Fig. 1). The antibacterials used in this study belong to the eight functional classes: beta-lactams (penicillins, cephalosporins, and carbapenems), tetracyclines, fluoroquinolones, phenicoles, aminoglycosides, polymyxins, sulfonamides, and nitrofurans. According to Magiorakos et al. [25] many strains under study were categorized as multi drug resistant (MDR) pathogens because of 98.9 % *K.* *pneumoniae* and 100 % *P.* *mirabilis* isolates were resistant to three or more functional classes of drugs simultaneously (Fig. 2). It should be noted that a large part of both *K.* *pneumoniae* (35.9 %) and *P.* *mirabilis* (54.8 %) strains were resistant to seven functional antibacterial classes. One *E.* *aerogenes* isolate was estimated as an extremely drug resistant because of resistance to all used antibiotics except TGC; three isolates were resistant to cefalosporins, carbapenems and NIT; two isolates were resistant to NIT only. Among *E.* *cloacae* isolates one isolate was MDR with sensitivity to IPM and AMK only; other isolates were resistant only to penicillins and cephalosporins.

### Resistance genotypes

**Fig. 3:**
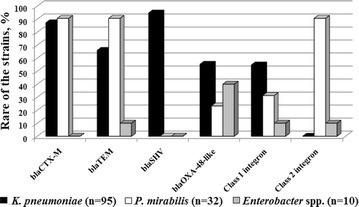
The proportion of bacterial isolates those are carrying the antibacterial resistance genes: *bla*_CTX-M_, *bla*_TEM_, *bla*_SHV_, *bla*_OXA-48-like_, class 1 and class 2 integrons

Detection of the *bla*_CTX-M_, *bla*_TEM_, *bla*_SHV_, *bla*_OXA-48-like_, *bla*_NDM_, and *bla*_KPC_ beta-lactamase genes as well as class 1 and class 2 integrons in the genomes of the strains has been done for study of the resistance molecular genetic mechanisms (Fig. 3). The *bla*_CTX-M_ genes were detected in 87.2 % *K.* *pneumoniae* and 90.6 % *P.* *mirabilis* isolates. Among 38 *bla*_CTX-M_ genes major (n = 36) were identified by sequencing as pandemic cephalosporinase *bla*_CTX-M-15_ gene and two *bla*_CTX-M-3_ genes. The *bla*_SHV_-type genes were detected only in the *K.* *pneumoniae* strains. No *bla*_NDM_ and *bla*_KPC_ genes have been obtained in our study. The *bla*_OXA-48-like_ carbapenemase genes were detected in 55.3 % of *K.* *pneumoniae*, in 23.3 % of *P.* *mirabilis*, and in 20.0 % of *Enterobacter* spp. isolates. DNA sequencing of 28 *bla*_OXA-48-like_ genes revealed two alleles: the *bla*_OXA-48_ gene was detected in *K.* *pneumoniae* (n = 17), *P.* *mirabilis* (n = 6) and *E.* *cloacae* (n = 1); the *bla*_OXA-244_ gene was detected in *K.* *pneumoniae* (n = 4), *P.* *mirabilis* (n = 1) and *E.* *aerogenes* (n = 1).

Mobile genetic elements, class 1 integrons were detected in 54.8 % *K.* *pneumoniae* and in 31.3 % *P.* *mirabilis* isolates (Fig. 3). Variable regions of class 1 integrons identified in *K.* *pneumoniae* carried three types of the gene cassette array: (*dfrA1*-*orfC*), (*dfrA17*-*aadA5*), and (*dfrA12*-*orfF*-*aadA2*). The class 1 integrons detected in *P.* *mirabilis* carried only one type of the gene cassette array (*dfrA17*-*aadA5*). Class 2 integrons obtained in *P.* *mirabilis* (90.6 %) isolates carried a gene cassette array (*dfrA12*-*sat2*-*aadA1*). However, one class 1 integron and one class 2 integron revealed in *E.cloacae* and *E.cloacae* isolates respectively did not have any gene cassette arrays, that might be considered as a reserve for future accumulating of the resistance genes. The integron identification numbers have been obtained in accordance with the nomenclature described by Moura et al. [26] using a specialized database INTEGRAL. The (*dfrA1*-*orfC*) gene cassette array was attributed to the In263 integron, the (*dfrA17*-*aadA5*) array to the In392–395 integron, the (*dfrA12*-*orfF*-*aadA2*) array to the In27 integron, and the (*dfrA12*-*sat2*-*aadA1*) array to the In2–4 integron. According to the report of Partridge et al. from the University of Sydney, Australia, the vast majority of gene cassettes submitted into the GenBank database to date are extremely rare (presented less than 10 times), while a small group of gene cassettes are extremely frequent, they were named “successful” cassettes [27]. All gene cassettes identified in clinical isolates collected from the ICU in our study belong to this group of “successful” cassettes. Thus, on the date Feb 6, 2015, the *aadA1* cassette has been presented in the GenBank database 910 times, the *aadA2* cassette 601 times, the *aadA5* cassette 248 times, the *dfrA1* cassette 348 times, the *dfrA12* cassette 245 times, the *orfF* cassette 218 times, the *dfrA17* cassette 209 times, the *orfC* cassette 135 times, and the *sat2* cassette 79 times. These data suggest the high prevalence of the gene cassettes detected in our study among bacterial pathogens.

Since the permeability of bacterial cell wall is important for multi drug resistance [23], we carried out the detection of porin gene *ompK36* and obtained this gene in 92.4 % *K.* *pneumoniae* isolates.

### Diversity of RAPD-genotypes

Great genetic heterogeneity of the pathogen populations was shown by RAPD-PCR genotyping for *K.* *pneumoniae* (n = 72) and *P.* *mirabilis* (n = 32). It was revealed 36 *K.* *pneumoniae* genotypes and 8 *P.* *mirabilis* genotypes. Four *K.* *pneumoniae* genotypes were prevalent in different periods of the study. The K23 genotype (n = 14) was major in 2013, while the K26 genotype (n = 5) was major in the first half of 2014, and the K30 (n = 6) and the K31 (n = 5) genotypes were prevalent in the second half of 2014. Interestingly, *K.* *pneumoniae* isolates belonged to K23, K26 and K31 genotypes carried the *bla*_OXA-48-like_ genes, whereas isolates belonged to K30 genotype did not have these genes. Among all *P.* *mirabilis* isolates collected in our study, the P1 RAPD-PCR-genotype (n = 23) was prevalent. It is noteworthy that all *P.* *mirabilis* isolates carried *bla*_OXA-48-like_ genes belonged to this prevalent genotype (Table 1). So, great variety of *K.* *pneumoniae* and *P.* *mirabilis* genotypes indicates no single source for dissemination of these pathogens. Perhaps presence of *bla*_OXA-48-like_ genes in bacterial genome provides some populational advantage for certain genotypes. Two RAPD-genotypes (E1 and E2) were identified for *E.* *aerogenes* isolates, but all three *bla*_OXA-244_-positive isolates were attributed to one genotype E1. Only one RAPD-genotype (E3) was determined for all *E.* *cloacae* isolates, including *bla*_OXA-48_-positive one.

**Table 1:** The features of *K. pneumoniae*, *P. mirabilis* and *Enterobacter *spp. isolates that were collected from the patients of the ICU in 2013–2014

Microbe	Strain	Isolation date^a^	Isolation source	Patient	*bla*_TEM_	*bla*_SHV_	*bla*_CTX-M_	*bla*_OXA-48-like_	*int1*	*ins1*	*int2*	*ins2*	Antibacterial resistance	RAPD type
*K. pneumoniae*	B-38	15.01.13	U	A	+	+	+	*bla*_OXA-48_	+	+	–	–	Pn Cf Cp Tc Qn Cm Su Nf	K7
*K. pneumoniae*	B-500	24.04.13	EA	B	+	+	+	*bla*_OXA-244_	+	**–**	–	–	Pn Cf Tc Qn Cm Su Nf	K23
*K. pneumoniae*	B-567	21.05.13	U	C	+	+	+	*bla*_OXA-244_	+	**–**	–	–	Pn Cf Cp Qn Su Nf	K23a
*K. pneumoniae*	B-600	21.05.13	EA	K	+	+	+	−	−	−	−	−	Pn Cf Qn Ag Su Nf	K29
*K. pneumoniae*	B-757 K	13.06.13	U	C	+	+	+	*bla*_OXA-244_	+	−	−	−	Pn Cf Cp Tc Qn Cm Ag Su Nf	K23
*K. pneumoniae*	B-811 K	16.07.13	IS	D	+	+	+	*bla*_OXA-244_	+	+	−	−	Pn Cf Cp Qn Su Nf	K1
*K. pneumoniae*	B-1667	31.10.13	U	K	+	+	+	*bla*_OXA-48_	−	−	−	−	Pn Cf Qn Cm Ag Su Nf	K29
*K. pneumoniae*	B-1896	12.12.13	U	K	+	+	+	*bla*_OXA-48_	−	−	−	−	Pn Cf Cp Tc Qn Cm Ag Su Nf	K29
*K. pneumoniae*	B-481/14	25.03.14	U	E	−	+	+	*bla*_OXA-48_	+	−	−	−	Pn Cf Cp Qn Cm Ag Nf	K23
*K. pneumoniae*	B-617/14	25.04.14	U	E	−	+	+	*bla*_OXA-48_	+	−	−	−	Pn Cf Cp Qn Cm Ag Nf	K23
*K. pneumoniae*	B-690/14 K	06.05.14	U	E	−	+	+	*bla*_OXA-48_	+	−	−	−	Pn Cf Cp Qn Cm Ag Nf	K23
*K. pneumoniae*	B-767/14	27.05.14	EA	N	−	+	+	*bla*_OXA-48_	+	+	−	−	Pn Cf Cp Tc Qn Cm Ag Su Nf	K28
*K. pneumoniae*	B-941/14	24.06.14	EA	F	+	+	−	*bla*_OXA-48_	+	−	−	−	Pn Cf Cp Tc Qn Cm Ag Su Nf	K36
*K. pneumoniae*	B-R1a	10.07.14	RS	F	−	+	+	*bla*_OXA-48_	+	+	−	−	Pn Cf Cp Tc Cm Ag Su	K31
*K. pneumoniae*	B-1699/14 K	23.10.14	EA	L	−	+	+	*bla*_OXA-48_	−	−	−	−	Pn Cf Cp Qn Cm Ag Nf	K31
*P. mirabilis*	B-123	07.02.13	U	G	+	−	+	−	+	+	+	+	Pn Cf Cp Qn Cm Ag Su Nf Co	P3
*P. mirabilis*	B-239	27.02.13	EA	H	+	−	+	−	+	+	+	+	Pn Cf Cp Qn Cm Ag Su Nf Co	P1_1_
*P. mirabilis*	B-371	28.03.13	U	E	+	−	+	−	−	−	+	+	Pn Cf Cp Qn Cm Nf Co	P1_1_
*P. mirabilis*	B-431 M	08.04.13	EA	H	+	−	+	*bla*_OXA-48_	+	+	+	+	Pn Cf Cp Qn Cm Ag Su Nf Co	P1_1_
*P. mirabilis*	B-757 M	13.06.13	U	C	+	−	+	*bla*_OXA-244_	−	−	+	+	Pn Cf Cp Tc Qn Cm Ag Nf	P1_1_
*P. mirabilis*	B-1939	17.12.13	W	M	+	−	+	−	−	−	+	+	Pn Cf Cp Qn Cm Ag Su Nf	P6
*P. mirabilis*	B-416/14	13.02.14	U	E	+	−	+	*bla*_OXA-48_	−	−	+	−	Pn Cf Cp Tc Qn Cm Nf	P1_1_
*P. mirabilis*	B-690/14 M	06.05.14	U	E	+	−	+	*bla*_OXA-48_	−	−	+	+	Pn Cf Cp Tc Qn Cm Nf	P1_1_
*P. mirabilis*	B-712/14 M	12.05.14	U	E	+	−	+	−	−	−	+	+	Pn Cf Tc Qn Cm Nf	P1_2_
*P. mirabilis*	B-682/14	06.05.14	EA	I	+	−	+	−	+	+	+	+	Pn Cf Cp Tc Qn Cm Ag Su Nf	P1_2_
*P. mirabilis*	B-799/14	26.05.14	EA	I	+	−	+	*bla*_OXA-48_	+	+	+	+	Pn Cf Cp Tc Qn Cm Ag Su Nf	P1_2_
*P. mirabilis*	B-828/14	02.06.14	EA	I	+	−	+	*bla*_OXA-48_	+	+	+	+	Pn Cf Cp Tc Qn Cm Ag Su Nf	P1_2_
*P. mirabilis*	B-1578/14	01.09.14	U	J	+	−	+	−	−	−	+	−	Pn Cf Cp Tc Qn Cm Ag Su Nf	P1_5_
*P. mirabilis*	B-1672/14 M	20.10.14	U	J	+	−	+	*bla*_OXA-48_	−	−	+	−	Pn Cf Cp Tc Qn Cm Ag Su Nf	P1_5_
*E. aerogenes*	B-2012/14	17.06.14	L	F	+	−	−	*bla*_OXA-244_	−	−	−	−	Pn Cf Cp Nf	E1
*E. aerogenes*	B-2137/14E	25.06.14	L	O	−	−	−	*bla*_OXA-244_	−	−	−	−	Pn Cf Cp Tc Qn Cm Ag Su Nf	E1
*E. aerogenes*	B-2212/14	30.06.14	L	F	−	−	−	*bla*_OXA-244_	−	−	−	−	Pn Cf Cp Nf	E1
*E. cloacae*	B-1530/14	30.08.14	U	N	−	−	−	*bla*_OXA-48_	+	−	−	−	Pn Cf Cp Tc Qn Cm Ag Su Nf	E3

*EA* endotracheal aspirate, *IS* intracranial sinus, *L* liquor, *RS* rectal swab, *U* urine, *W* wound, *A*, *B*, *C*, *D*, *E*, *F*, *G*, *H*, *I*, *J*, *K*, *L*, *M*, *N* and *O* patients, *bla*_OXA-244_, *bla*_CTX-M_, *bla*_TEM_, *bla*_SHV_ beta-lactamase genes, *int1* class 1 integrase gene, *ins1* genetic cassette array of class 1 integron, *int2* class 2 integrase, *ins2* genetic cassette array of class 2 integron, *Pn* penicillins, *Cf* cephalosporins, *Cp* carbapenems, *Tc* tetracyclines, *Qn* fluoroquinolones, *Cm* phenicols, *Ag* aminoglycosides, *Su* sulfonamides, *Nf* nitrofurans, «+» positive, «−» negative

^a^Day, month, year

### Spread of *bla*_OXA-48-like_ genes in ICU

**Fig. 4:**
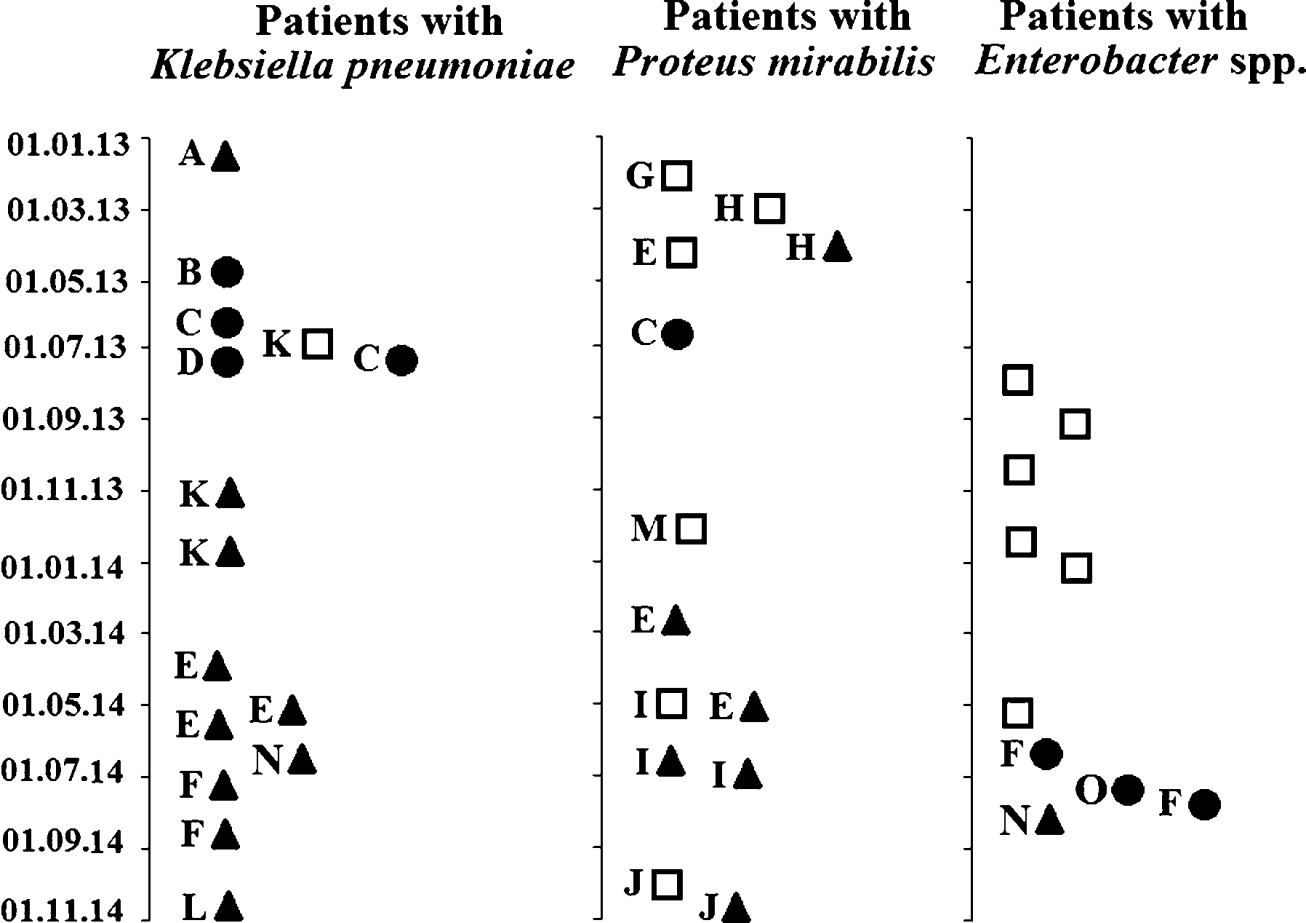
Emergence of *K.* *pneumoniae*, *P.* *mirabilis* and *Enterobacter *spp. clinical isolates carrying *bla*_OXA-48-like_ genes on the period from 01.01.2013 to 01.11.2014: *square* no gene, *filled triangle* *bla*_OXA-48_ gene, *filled circle* *bla*_OXA-244_ gene; *A*, *B*, *C*, *D*, *E*, *F*, *G*, *H*, *I*, *J*, *K*, *L*, *M*, *N* and *O* patients

The case of the *bla*_OXA-48_ gene acquisition by *K.* *pneumoniae* within ICU was demonstrated for the Patient K. The first isolate belonged to K29 genotype (May 21, 2013) had no the *bla*_OXA-48_ gene; the next two isolates of K29 genotype (Oct 31, 2013 and Dec 12, 2013) have already acquired the *bla*_OXA-48_ gene (Table 1; Fig. 4). The first case of the *bla*_OXA-48_ gene emergence in a new bacterial host, *P.* *mirabilis*, was registered in the Patient H (Apr 08, 2013). It was happened four months after the start of the study, while such genes in *K.* *pneumoniae* isolates were detected during the entire two year period. Two months later, Jun 13, 2013, the *bla*_OXA-244_ gene was identified in *P.* *mirabilis* isolate collected from the Patient C. Unfortunately, a retrospective epidemiological analysis has not allowed concluding relatedness of occurrence the *bla*_OXA-244_ gene in Russia and in Spain where this gene was first described in 2013 [13]. The first case of the *bla*_OXA-244_ gene emergence in a bacterial host *E.* *aerogenes* was registered in the Patient F (Jun 17, 2014), i.e. 18 months after the start of the study. The first case of the *bla*_OXA-48_ gene identification in *E.* *cloacae* was obtained in the Patient O (Aug 30, 2014), i.e. 20 months after the start of the study. So, two *bla*_OXA-48-like_ alleles have disseminated in the ICU simultaneously.

Comparative analysis of two or more *P.* *mirabilis* isolates collected from the same patient has been done for four patients: E, H, I, and J. All of these patients had no the *bla*_OXA-48-like_ gene in their first isolates. The second isolates (Patients H and J) and the second and the third isolates (Patients E and I) carried the *bla*_OXA-48-like_ genes. So, the *bla*_OXA-48-like_ genes acquisition by *P.* *mirabilis* occurred in the ICU, presumably from *bla*_OXA-48-like_-positive *K.* *pneumoniae* which is half of all *K.* *pneumoniae* isolates under study. Additional proof of this hypothesis is the concurrent isolation of *bla*_OXA-244_-positive *K.* *pneumoniae* and *P.* *mirabilis* from urine of the Patient C (Fig. 4), as well as isolation of *bla*_OXA-48_-positive *K.* *pneumoniae* and *E.* *cloacae* from endotracheal aspirate and urine correspondently of the Patient N. Unfortunately, such clear hypothesis cannot to explain the fact of the *bla*_OXA-48_-positive *K.* *pneumoniae* isolation from endotracheal aspirate and feces of the Patient F and—on the same period—the *bla*_OXA-244_-positive *E.* *aerogenes* isolates from liquor of this patient (Table 1).

### Plasmid localization of *bla*_OXA-48-like_ genes

It is known that the *bla*_OXA-48-like_ genes localized on the IncL/M conjugative plasmids [11, 22]. PCR-detection of the specific IncL/M plasmid markers—*repA* and *traU*—showed the presence of the plasmid in all *bla*_OXA-48-like_-positive *P.* *mirabilis* (n = 7) and *Enterobacter* spp. (n = 4) isolates, and the absence of such plasmid in the *bla*_OXA-48-like_-negative isolates. The same correlation between *bla*_OXA-48-like_ gene and the markers of the IncL/M plasmid was revealed for *K.* *pneumoniae* strains. So the *bla*_OXA-48-like_ genes in bacterial isolates under study were located on IncL/M conjugative plasmids.

The acquisition of the *bla*_OXA-48-like_ gene consisting plasmid IncL/M has been demonstrated by the experimental conjugation at *intra*- and *inter*-species transfer. Two clinical *K.* *pneumoniae* strains, B-500 and B-757 K, resistant to cefotaxime were used as donors. The *K.* *pneumoniae* M-9 Rif strain was used as a recipient at intraspecies conjugation, and the *E.* *coli* HB101 Rif was used as a recipient at interspecies conjugation. Selection of transconjugants was done by selective markers cefotaxime and rifampicin. The efficiency of conjugation was 2.0–8.5 × 10^−4^ at *intra*- and 2.0–3.0 × 10^−3^—at *inter*-species conjugation. The IncL/M transfer from the donor to the recipient was detected by the plasmid markers *repA* and *traU* appearance in the transconjugants. The *bla*_CTX-M_, *bla*_TEM_, *bla*_SHV_, and class 1 integrase genes were detected in the transconjugant cells (Table 2). The distribution of genetic markers in the transconjugants shows that both donor *K.* *pneumoniae* strains carried two conjugative plasmids: the IncL/M plasmid (*bla*_OXA-244_^+^*repA*^+^*traU*^+^) and the unidentified plasmid (*bla*_CTX-M_^+^*bla*_TEM_^+^*bla*_SHV_^+^) (Table 2).

**Table 2:** Donor, recipient and transconjugant *K.* *pneumoniae* and *E.* *coli* strains features at *intra*- and *inter*-species conjugation

Role at conjugation	Strain	Conjugation effectiveness	Genetic markers								Plasmids
			*repA*	*traU*	*bla*_OXA-244_	*bla*_CTX-M_	*bla*_TEM_	*bla*_SHV_	*int1*	*ompK36*	
Donor	*K.* *pneumoniae* B-500		+	+	+	+	+	+	+	+	L/M, UP
Donor	*K.* *pneumoniae* B-757 K		+	+	+	+	+	+	+	+	L/M, UP
Recipient	*K.* *pneumoniae* 9 Rif		−	−	−	−	−	+	−	+	−
Recipient	*E.* *coli* HB-101 Rif		−	−	−	−	−	−	−	−	−
Transconjugant	*K.* *pneumoniae* 9/500-1	2.0 × 10^−4^	+	+	+	+	+	+	+	+	L/M, UP
Transconjugant	*K.* *pneumoniae* 9/500-2	2.0 × 10^−4^	−	−	−	+	+	+	+	+	UP
Transconjugant	*E.* *coli* HB/500-1	2.0 × 10^−3^	+	+	+	−	−	−	−	−	L/M
Transconjugant	*K.* *pneumoniae* 9/757-1	8.5 × 10^−4^	+	+	+	+	+	+	+	+	L/M, UP
Transconjugant	*K.* *pneumoniae* 9/757-2	8.5 × 10^−4^	−	−	−	+	+	+	+	+	UP
Transconjugant	*E.* *coli* HB/757-1	3.0 × 10^−3^	+	+	+	−	−	−	−	−	L/M

*repA*, *traU* genetic markers of the IncL/M plasmid, *bla*_*OXA-244*_, *bla*_*CTX-M*_, *bla*_*TEM*_, *bla*_*SHV*_ beta-lactamase genes, *int1* class 1 integrase gene, *ompK36 K.* *pneumoniae* porin protein gene, «+» positive, «−» negative, *L/M* conjugative plasmid IncL/M, *UP* unidentified conjugative plasmid

## Conclusions

The molecular genetic mechanisms for multi-drug resistance of *K.pneumoniae*, *P.mirabilis*, *E.aerogenes* and *E.* *cloacae* isolates collected from a Moscow neurosurgical ICU in 2013–2014 were shown associated with *bla*_CTX-M_, *bla*_TEM_, *bla*_SHV_ beta-lactamase genes, class 1 and class 2 integrons, as well as with *bla*_OXA-48-like_ carbapenemase genes. Two alleles genes, the *bla*_OXA-48_ and the *bla*_OXA-244_, have been identified in the isolates under study. Moreover, *P.* *mirabilis*, *E.* *aerogenes* and *E.* *cloacae* are first determined as bacterial hosts for the of *bla*_OXA-48-like_ genes in Russia. The *bla*_OXA-48-like_ genes were found to be integrated into the well-known conjugative plasmids of IncL/M incompatibility group that probably were transferred from *K.* *pneumoniae* to *P.* *mirabilis* and to *E.* *cloacae* during the period of the patient stay in the ICU. The source of the *bla*_OXA-244_ gene acquired by *E.* *aerogenes* isolates and the time of this event were not recognized.

So, this study highlights ongoing expanding of CPE pathogens in Russia. Obtained knowledge is important for an adequate evaluation of the epidemiological situation, for predicting the development of the situation in the future, and for the correct choice of the optimal strategy for the antibacterial therapy.
